# Arthroscopic Reduction and Fixation With a Knotless Double-Row Construct Provides Good Results for Displaced Greater Tuberosity Fractures

**DOI:** 10.1016/j.asmr.2020.10.014

**Published:** 2021-03-11

**Authors:** Mohammad Bahman, Vanessa Costil, Mathilde Gaume, Marc-Antoine Rousseau, Patrick Boyer

**Affiliations:** a University of Paris, Paris, France; b Clinic of Franciscaines Ramsay, Versaille, France

## Abstract

**Purpose:**

The purpose of the study is to describe the functional and structural outcomes of the arthroscopic 4-strand, knotless, double-row construct with suture tapes for the surgical treatment of displaced and/or comminuted greater tuberosity fractures of the humerus.

**Methods:**

Patients were enrolled between December 2012 and January 2018. The main inclusion criteria were a comminuted and/or displaced tuberosity fracture with a displacement of at least 5 mm in any plane fixed under arthroscopy using a 4-strand, knotless, double-row construct. The technique involves reducing the displaced fragment with 2 medially placed, transtendinous anchors and compressing the greater tuberosity using the tapes from these medial anchors in 2 laterally placed anchors. The exclusion criteria were a fracture that was more than 10 days old at the time of surgery or a history of shoulder surgery and 3- or 4-part fractures. The postoperative rehabilitation protocol was similar for all patients. Constant scores, Quick Dash, return to work and sport, and complications were reported after a minimum follow-up period of 24 months. Bone healing was systematically evaluated on standardized radiographs, including lateral scapula view and anteroposterior views.

**Results:**

Twenty-one patients were enrolled in this study. One patient did not complete the follow-up examination period and thus was excluded, leaving 20 patients in this study. At a median (SD) follow-up of 32 (9) months, the median (SD) Constant score was 94.7 (7.3) points, the median (SD) Quick Dash was 1.7 (4) points, and median (SD) visual analog scale score was 0.5 (1.4). All patients returned to previous work and sport level. No malunions or nonunions were seen. One conversion to open surgery was required for failure of the lateral row during surgery in a 62-year-old woman with osteopenic bone. Two patients experienced complex regional pain syndrome in the postoperative period that resolved after nonoperative treatment.

**Conclusions:**

In this series, the use of arthroscopy combined with the biomechanical properties of knotless double-row constructs contributed to postoperative satisfactory functional results and healing of greater tuberosity fracture. In addition, range of motion was early, and no hardware removal was required. However, care should be taken with osteopenic bone where anchorage can fail.

**Level of Evidence:**

Level IV, case series.

Greater tuberosity fractures are described as avulsion fractures of the rotator cuff and have been proved to have a substantial impact on the shoulder functional outcome.^1^^,^^2^ Open reduction and internal fixation are recommended when the greater tuberosity is displaced more than 0.5 cm based on anatomic, biomechanical, and clinical studies.^3^ Indeed, nonoperative treatment for these displaced fractures could lead to nonunions, limited range of motion in abduction and external rotation, and impingement between the greater tuberosity and the acromion.^4^

These fractures are known to be challenging to treat, as the small size of the fragments and the comminution of the fracture often prevent the use of conventional techniques of internal fixation to achieve adequate reduction and stable fixation.^5^^,^^6^

In a continuous attempt to improve the results after arthroscopic rotator cuff repair, a new arthroscopic technique—suture bridging—has been developed. It aims at increasing the amount of native footprint covered by the repaired tendon to create a larger tendon-bone interface to improve healing.^7^, ^8^, ^9^ Initially based on a 2 medial mattress suture configuration with 4 tied suture bridges fixed laterally by knotless anchors, the procedure has shifted recently to a 4-strand, knotless, double-row construct with suture tapes of 2-mm width.^10^, ^11^, ^12^ The new construct, compared with the suture-bridge technique, should enhance footprint coverage and maximize compression of the tendon-bone interface by the suture tapes.^12^

The purpose of the study is to describe the functional and structural outcomes of the arthroscopic 4-strand, knotless, double-row construct with suture tapes for the surgical treatment of displaced and/or comminuted greater tuberosity fractures of the humerus.

We hypothesized that this construct can provide a strong fixation, leading to the healing of the fracture and subsequent satisfactory functional outcome.

## Methods

Patients who had a displaced and comminuted fracture of the greater tuberosity were consecutively enrolled in this study between December 2012 and January 2018. Data were recorded prospectively and then analyzed retrospectively by an independent observer.

Informed consent was obtained from all patients. Described research adhered to the tenets of the Declaration of Helsinki. The institutional review board considered this retrospective study as "standard care" and did not require further reviews.

Patient records were reviewed for the following inclusion criteria: displacement of the tuberosity of at least 5 mm in any plane, arthroscopic reduction, and fixation using a 4-strand, knotless, double-row construct.

Patients who had a fracture that was more than 10 days old at the time of surgery or a history of shoulder surgery were excluded. Patients with 3- or 4-part fractures were also excluded.

Radiographs in 2 planes (anteroposterior and lateral views) were used to analyze the fracture and the displacement of the tuberosity. Comminution was defined as 2 or more fragments of the greater tuberosity bone fragment attached to the rotator cuff tendons, as observed on radiographs. Radiographic assessment was completed by either a preoperative computed tomography scan or magnetic resonance imaging by a specialized radiologist. In addition, arthroscopic observation always confirmed the presence of a rotator cuff tear (supraspinatus tendon) associated with the tuberosity fracture.

All patients were operated on by a single surgeon using the same surgical technique described below.

### Surgical Technique

**Fig 1:**
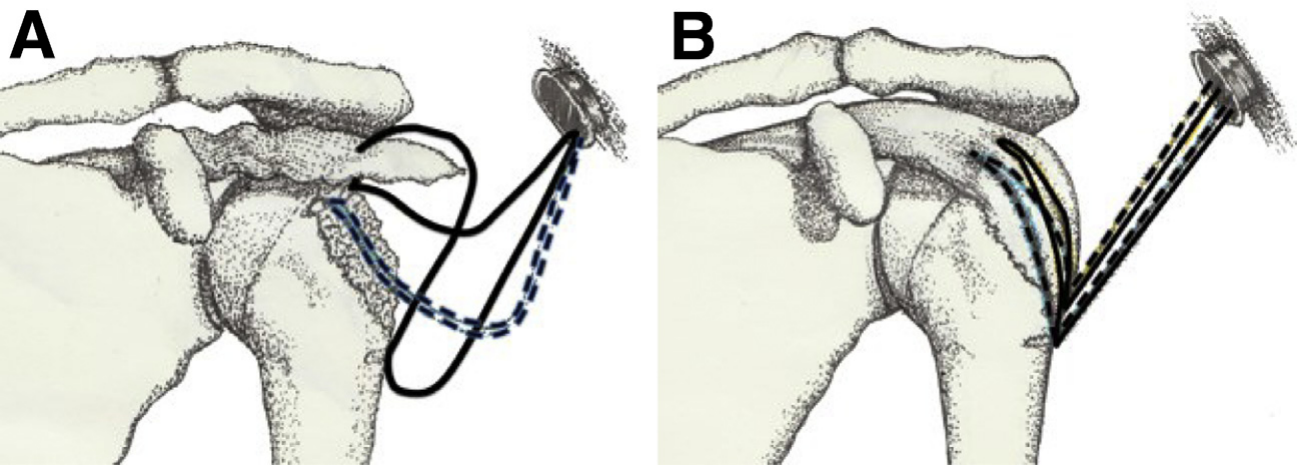
Drawing showing the principles of the arthroscopic fixation on a left shoulder.

The procedure was realized under general anesthesia in a beach-chair position without traction. We used 5 portals, the standard posterior and lateral viewing portals, anterolateral and posterolateral instrumental portals, and an anteriosuperior portal (that allowed us to create holes and insert screws for the lateral row at the articular margin). Principal of arthroscopic fixation (Fig 1A and B). In 1 case, because of the big size of the fragment, we used an additional lateral portal lower than the other to insert the anchors of the lateral row.

The first step was the intra-articular exploration using the standard posterior viewing portal. In every case, the exploration confirmed the existence of a comminuted displaced fracture with rotator cuff avulsion (supraspinatus tendon). Associated injuries were systematically evaluated. The anterolateral portal was then realized to introduce a shaver through the fractures following the direction of a needle. The shaver was used to clean the joint by evacuating the fracture hematoma.

**Fig 2:**
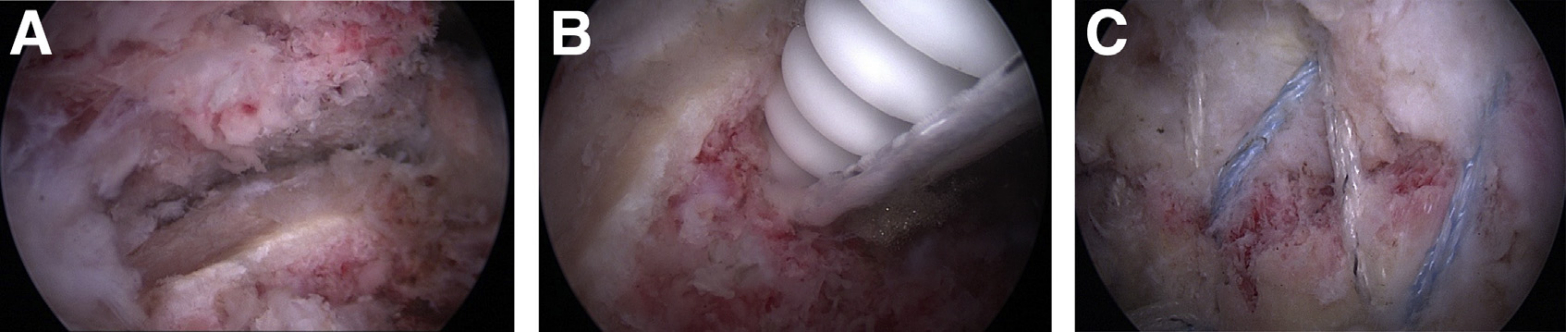
(A) Lateral arthroscopic view of the displaced greater tuberosity fractures of the right shoulder. (B) Insertion of the medial anchorage with the tape. (C) Final arthroscopic view of the fracture fixed using the knotless double-row construct.

Then, the arthroscope was retrieved from the joint and inserted into the subacromial space using the posterior portal. Again, using the shaver, bursa and hematoma were removed. Acromioplasty or acromioclavicular sectioning was never performed. When the view was perfectly clear, reduction of the fracture was successfully tested using a grasper. At this point, to obtain a better view, a lateral portal for the arthroscope was created with a needle under the control of the posterior view and then a posterolateral portal (Fig 2A).

The knotless, self-reinforcing, double-row repair was undertaken with braided suture tapes. First, two 4.75-mm bioabsorbable anchors (SwiveLock C; Arthrex) preloaded (into the eyelet) with 2-mm-wide tapes (FiberTape and TigerTape; Arthex) were placed medially 1 to 2 mm lateral to the articular margin. After a pilot hole was made, the anchors were advanced until their body made contact with the bone. Then, the driver handle was malleted directly to fully insert the anchor body until it was flush with the bone. Anchors insertion was systematically tested (Fig 2B).

Both limbs of tape from each of the 2 anchors were passed through the supraspinatus tendon around 10 mm medial to the lateral edge of the rotator cuff tear with a suture passer (Scorpion; Arthrex). No knot was tied. Each tape tail from each medial anchor was retrieved with a grasper and threaded through each of the 2 lateral anchors. A bone socket was prepared with a punch from 5 to 10 mm lateral to the edge of the tuberosity fracture. Then, the SwiveLock anchors were inserted to create a 4-bar interconnected construct once adequate tension was set. The repair was always performed in the same manner regardless of the size of the fragment of the greater tuberosity except in 2 cases where the fragment was very large and we inserted 3 anchors medially and laterally.

Reduction of the fracture was obtained using the anchor driver as the reduction tool. At the end of the procedure, the shoulders were mobilized to detect unsatisfactory fixation under arthroscopic control (Fig 2C).

In addition, 1 tenotomy for SLAP 4 in a 60-year-old was performed in association with fracture fixation. One Bankart repair for SLAP 2 and Bankart lesion was performed. In another case, we combined the procedure with a subscapularis repair.

### Postoperative Protocol

The shoulders were immobilized for 4 weeks in a sling (Sober). Shoulder rehabilitation consisted of free passive range-of-motion exercises starting from postoperative day 1. Active motion was initiated at 4 weeks. Rotator cuff–strengthening exercises began 10 to 12 weeks after surgery. Full return to sports and heavy labor were allowed after 3 months according to individual functional recovery.

### Clinical and Radiographic Assessment

Clinical and radiographic follow-up occurred at 4 weeks, 3 months, and every 6 months postoperatively until final follow-up by an independent observer. Clinical assessment included the following data: patient’s demographics, cause of injury, associated injuries, hand dominance, time to surgery, and range of motion. The Shoulder and Hand (QuickDash) score,^13^ the Constant-Murley score,^14^ and the visual analog scale (VAS) pain score were completed at final follow-up. Values were reported with 1 decimal place.

Time to return to work and to sports was assessed. Patients were asked to express global satisfaction on the surgical treatment by responding either “satisfied” or “not satisfied.” Any postoperative complication or failure was recorded, including nonunion, malunion, infection, or complex pain regional syndrome.

Lateral and anteroposterior radiographs of the glenohumeral joint were assessed to determine union and tuberosity-head relation.

## Results

The study group included 13 men and 8 women. The median age of the patients was 45.7 years (range, 20-62). Of the 21 patients with a displaced tuberosity fracture who were included in this study, 1 patient did not complete the follow-up examination period. Thus, the outcomes were determined for 20 patients.

The injury was the result of direct impact to the greater tuberosity in 16 patients and indirect in 5 patients. The direct impact included contact sports in 5 patients, motor vehicle or cycle accidents in 4 patients, and fall onto the point of the shoulder in 7 patients. The indirect mechanism in 5 patients included a hyperabduction–external rotation of the shoulder or resisted hyperabduction during a fall.

The dominant arm was affected in 14 patients. Median (SD) follow-up was 32 (9) months. Median delay between fracture and arthroscopic procedure was 5 (range, 2-7) days. The median duration of surgery was 90 (range, 60-150) minutes.

At final follow-up, the median (SD) QuickDash score, Constant score, and VAS score were, respectively, 1.7 (4; range, 0-11), 94.7 (7.3; range, 82-100), and 0.5 (1.4; range, 0-2).

### Combined Injuries

**Fig 3:**
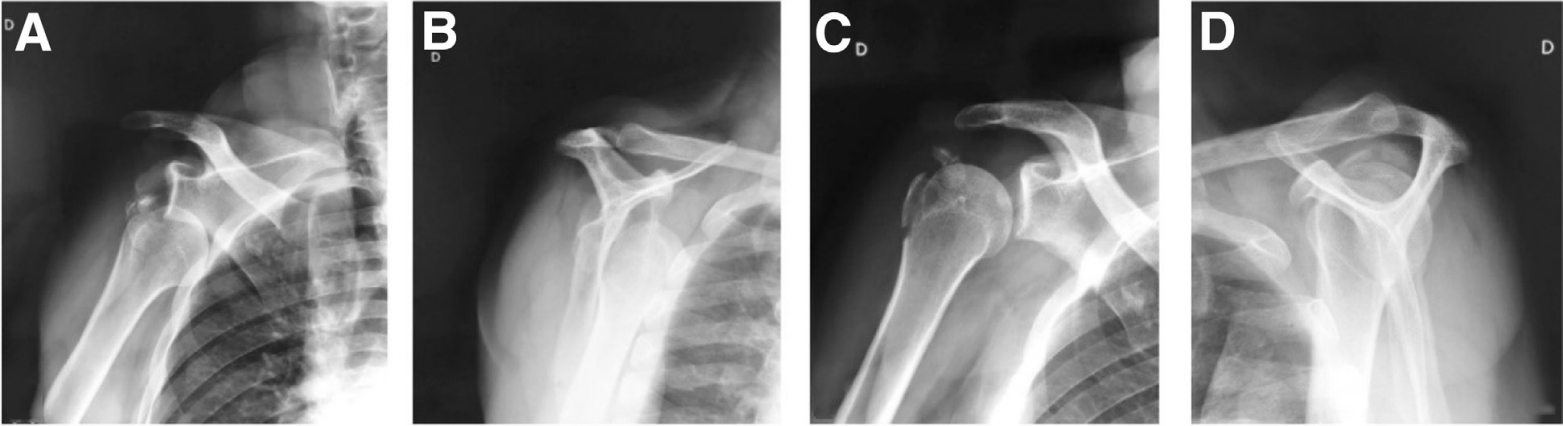
Displaced greater tuberosity fracture associated with anterior dislocation before (A, B) and after reduction (C, D) of a right shoulder.

Six patients sustained concomitant anterior glenohumeral dislocation, which was reduced before examination at our institute. When the fracture of the greater tuberosity was still displaced more than 5 mm, patients underwent surgery (Fig 3A-D). Combined lesions that were treated evolved positively, and no patient experienced recurrent anterior dislocation.

Patients were able to return to their previous job at a mean 11 (range, 8-14) weeks after surgery and to resume heavy manual labor at a mean 20 (range, 12-28) weeks after surgery. All patients had resumed their recreational sport activities (no competition activity) after an average time of 21 weeks after surgery. Eleven patients (85%) were satisfied with the functional outcome.

**Fig 4:**
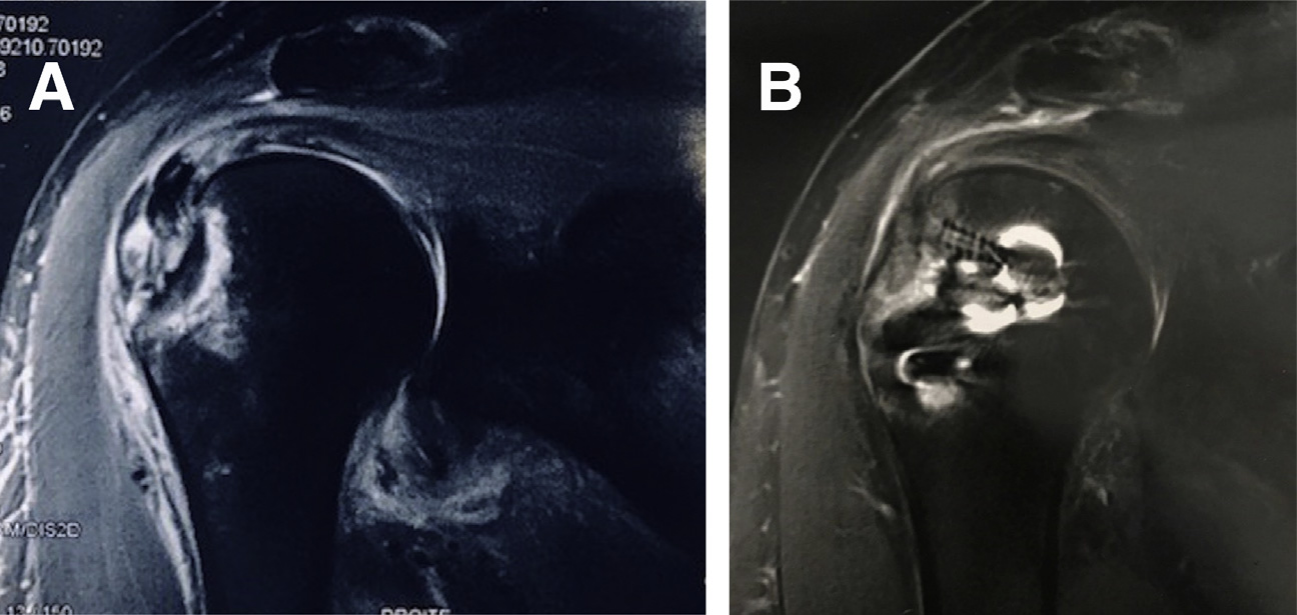
(A) Magnetic resonance imaging (MRI) frontal view of the right shoulder showing comminuted displaced fracture with the supraspinatus tendon. (B) At the last follow-up, MRI of the right shoulder shows healed cuff and greater tuberosity fracture.

Follow-up radiographs showed healing of the fracture in all cases (Fig 4 A and B).

## Complications

In the early postoperative period, 2 patients experienced complex pain regional syndrome that resolved spontaneously with prolonged physiotherapy. Restoration of their range of motion and pain relief were comparable to other patients in the last follow-up appointment.

In one case, conversion to open surgery was required due to failure of the lateral row at the moment of anchor insertion in the osteopenic bone of a 62-year-old woman. Transosseous sutures through the greater tuberosity fracture and the humerus were performed. In the last follow-up, healing was achieved, but this patient still complained of painful shoulder. One patient needed secondary surgery for hard for hardware removal.

## Discussion

In this series, arthroscopic fixation using a knotless double-row construct for greater tuberosity fractures confirms our hypothesis in achieving good to excellent outcomes in all cases.

This study describes the clinical and radiographic outcomes of a knotless double-row construct technique repair for greater tuberosity fractures.^15^

These results may be explained by the use of an arthroscopic procedure that avoids extensive dissection of the deltoid muscle, that has a low rate of postoperative complications, and that allows early rehabilitation for patients compared with an open procedure. Second, the biomechanical properties of the knotless double-row construct enable strong fixation and compression of the fragments even if these are comminuted and osteoporotic. In a biomechanical study comparing double-row and suture-bridge techniques to screw fixation, Lin et al.^16^ reported that suture anchors provided stronger fixation than screws. Suture anchor fixation was significantly better under cyclical load testing and ultimate load to failure.^16^ With the use of a knotless double-row construct, the 2 mm interconnected maximizes compression and fixation of the fragment and tissue cut-through resistance.^12^

Such results are in accordance with the few series in the literature that reported results using suture bridge for the fixation of such fractures.^2^^,^^17^

In his series, Ji et al.^17^ operated on 40 patients using an arthroscopic suture-bridge repair for the greater tuberosity fracture, with a mean follow-up of 32 months. In the last follow-up, functional scores and improvement of range of motion were achieved. Radiographic control showed minimal residual displacement. The main complication reported in 5 cases was anchor protrusion.^17^

Several investigators have reported the outcomes after different arthroscopic treatment of displaced greater tuberosity fractures. Ji and colleagues^18^ treated 16 patients with an arthroscopic double-row suture anchor fixation technique and had 3 excellent results, 11 good results, and 2 poor results according to the UCLA score at a mean follow-up of 2 years with a mean American Shoulder and Elbow Surgeons (ASES) score of 88.1. Tsikouris and colleagues^19^ reported outcomes in 12 athletes treated with arthroscopic reduction and single-row fixation. Six of these 12 patients were professional athletes, and all patients achieved complete radiographic fracture healing, with all athletes returning to their preinjury activity levels. Nevertheless, single arthroscopic fixation using double-row suture anchors could be associated with potential displacement because of the lack of compression of the tuberosity fragment.^1^

Other benefits exist when such fractures are treated arthroscopically. Greater tuberosity fractures, particularly those fractures seen in conjunction with anterior glenohumeral dislocations, often have associated intra-articular injuries, including Bankart lesions, loose bodies, labral tears, or cuff tear. These fractures are often associated with anterior glenohumeral dislocation ranging from 5% to 30%.

Ji et al.^17^ reported in their series that associated injuries occurred in 52.5% of the cases (21 patients), including 8 rotator cuff tears, 7 SLAP lesions, 3 Bankart lesions, and 3 glenoid rim fractures. These injuries are not fully appreciated and cannot be adequately addressed with open reduction of greater tuberosity fractures via traditional surgical approaches.^20^ In our series, associated injuries were observed in 2 cases and treated during the same procedure by 1 tenotomy/tenodesis and 1 Bankart repair. Another benefit of the arthroscopic approach over open surgery for these fractures is the avoidance of systematic hardware removal.

Arthroscopy has been also successfully reported in the management of proximal humerus malunion with tuberoplasty and rotator cuff retensioning.^21^, ^22^, ^23^ However, limits to this technique exist. In osteopenic patients, the risk of failure is high, especially during the lateral row fixation due to the high tension applied on the anchor. We observed this complication in 1 case in which the conversion to open surgery was done.

The size of the fragment is another limitation, especially when the fragment is big. For this reason, in 1 case, a lateral fixation at the junction between the metaphysis and the diaphysis was performed. This required drilling before inserting the anchor. In addition, suture bridging could be insufficient to compress the large fragment. Therefore, in such cases, fixation by plate or screws should be preferred. At the opposite, when the fragment measures 5 mm or less, some have recommended excision of the bone fragment and repair of the rotator cuff.^24^

The construct, despite only 2 suture-passing steps and no tied medial mattress, can be technically demanding. The fragment reduction and/or the management of the bleeding and hematoma is also challenging in some cases.

In addition, whatever the fixation (screws, plating, wire, or arthroscopic double row), it is important to treat these fractures early (before day 8) to avoid retraction of the rotator cuff in a posterior direction.

Special attention has to be paid to impingement of the biceps tendon by the medial edge of the tuberosity fragment or a foreign body reaction to the fixation material. These adverse events could result in severe, persistent pain with active motion in the rehabilitation period as previously reported with anchor fixation.^25^ Intraoperative confirmation of the reduction by palpation of the lateral region of the bicipital groove and by movement of the shoulder through the entire range of motion is essential to prevent postoperative bicipital impingement. When this complication happens, tenodesis or tenotomy of the biceps tendon with excision of the medial edge of the tuberosity should provide immediate and complete relief of pain.

### Limitations

The small sample size is the main limitation of this study. Another limitation was that surgeries were performed by a single surgeon, and thus the results obtained were related to that individual surgeon’s skills and experience.

## Conclusions

In this series, the use of arthroscopy combined with the biomechanical properties of knotless double-row constructs contributed to postoperative satisfactory functional results and healing of greater tuberosity fracture. In addition, range of motion was early and no hardware removal was required. However, care should be taken with osteopenic bone, in which anchorage can fail.

## Supplementary Data

ICMJE author disclosure forms
